# The national burden of road traffic injuries in Thailand

**DOI:** 10.1186/1478-7954-9-2

**Published:** 2011-01-18

**Authors:** Vallop Ditsuwan, Lennert J Veerman, Jan J Barendregt, Melanie Bertram, Theo Vos

**Affiliations:** 1 Faculty of Health and Sport Science, Thaksin University, Phatthalung Province, 93110, Thailand; 2 The University of Queensland, School of Population Health, Brisbane, Australia

## Abstract

**Background:**

This study quantifies the burden of road traffic injuries (RTIs) in Thailand in 2004, incorporating new Thai data on mortality and the frequency and severity of long-term disability.

**Methods:**

We quantified the uncertainty around national RTI mortality estimates based on a verbal autopsy study that was conducted to correct for the large proportion of ill-defined deaths in the vital registration system. The number of nonfatal RTI victims was estimated using hospital and survey data. We used the proportion and severity of long-term disabilities from a recent Thai study, instead of the standard Global Burden of Disease assumptions, to calculate the burden due to long-term disability. To evaluate changes over time, we also calculated the burden of RTIs in 2004 using the method and assumptions used in 1999, when standard Global Burden of Disease assumptions were used.

**Results:**

The total loss of disability-adjusted life years due to RTIs was 673,000 (95% uncertainty interval [UI]: 546,000-881,000). Mortality contributed 88% of this burden. The use of local data led to a significantly higher estimate of the burden of long-term disability due to RTIs (74,000 DALYs [95% UI: 55,400-88,500] vs. 43,000 [UI: 42,700-43,600]) using standard Global Burden of Disease methods. However, this difference constituted only a small proportion of the total burden.

**Conclusions:**

The burden of RTIs in 2004 remained at the same high level as in 1999. The use of local data on the long-term health consequences of RTIs enabled an estimate of this burden and its uncertainty that is likely to be more valid.

## Background

Road traffic injuries (RTIs) are an important public health problem worldwide, with the majority of RTIs occurring in low- and middle-income countries [1]. A survey in 2008 in 178 countries reported that 91% of deaths related to RTIs are in low- and middle-income countries, which contain only half of the world's registered vehicles [2]. A number of interventions have contributed to a significant reduction in the burden of RTIs in developed countries [3,4]. These interventions are generally implemented and evaluated in high-income countries, but they may also be effective in low- and middle-income countries [5]. In these countries, the first challenge is to assess and monitor the size of the RTI problem. Priority-setting for the prevention of RTIs requires accurate and reliable information on the disease burden RTIs cause [5-8]. Guidelines recommend using local data where these exist because the extent and nature of RTIs in each country varies depending on modes of transportation and traffic volumes [2,5,8]. In low- and middle-income countries, however, data sources are often of low quality, nonrepresentative, difficult to access, and contain a limited number of variables [5,7,8]. The magnitude of RTIs in terms of disability-adjusted life years (DALYs) is usually estimated using a standard set of assumptions on duration and severity of disability as well as the proportion of RTI victims who have long-term disabilities [6,9,10].

This study used the best available methods and evidence to calculate the burden of RTIs in Thailand, a low-/middle-income country. We updated the burden of RTIs first estimated for 1999 using Global Burden of Disease (GBD) methodology and assumptions [11]. The present study was carried out as part of the Setting Priorities based on Information on Cost-Effectiveness (SPICE) project, a collaboration between the Thai Ministry of Public Health and researchers from Thailand and the University of Queensland. The SPICE project carried out a cause of death study, verifying the Thai mortality structure, which we utilize in the current study [12]. In addition, we carried out a study in which we estimated the frequency and severity of long-term disabling outcomes of RTIs [13] (manuscript submitted to *Injury Prevention*). We used these local data in the present study. To compare the results to the estimate for 1999, we used standard GBD methods and assumptions in a parallel analysis.

## Methods

The burden of RTIs in Thailand in 2004 was calculated in disability-adjusted life years (DALYs). We separately determined and then summed the number of years of life lost (YLL) due to premature mortality from RTI events in 2004, and the years lost to disability (YLD) due to temporary or permanent disabilities arising from RTIs. We calculated the burden of RTIs by age group and sex. Future health was discounted at a rate of 3% per year. We decided not to use the GBD age-weighting function, which is not universally accepted [14] and was not used in the 1999 Thai Burden of Disease study to which we intended to compare our results [15], nor is it used in the cost-effectiveness analysis that we planned to conduct in further work. The models were implemented in Microsoft Excel (Microsoft Corp., Redmond, WA, USA).

### Fatal RTI victims

In order to calculate YLL, we needed to determine age- and sex-specific RTI mortality rates in 2004. Approximately 60-65% of deaths registered in Thailand are coded to specific causes of death, while the remainder are assigned to ill-defined codes [16]. In this study, we used the total number of fatal RTIs estimated by the SPICE cause of death study [17]. The cause of death study was undertaken in a representative sample of 11,984 deaths from the 2005 vital registration data. For each death record, the cause of death was ascertained from two sources. The first source was a household interview with a close relative of the deceased using a standardized verbal autopsy (VA) tool. For 9,819 (82%) of the 11,984 deaths, a VA interview was completed, and a cause of death was assigned. The second source of causes of death was from a medical records review for those who died at a health facility where they had been treated prior to death. Of the 11,984 deaths, the interviews ascertained that 4,644 deaths took place in a hospital, of which 3,316 cases (71.2%) had adequate information in their medical records to be assigned a "certified cause of death" by medical experts. The redistribution of causes of death more than doubled the number for hospital deaths and almost doubled the number of home deaths assigned to RTIs. In both cases, the specificity of the vital registration system was high, but sensitivity was low. More detail on the estimates of mortality for Thailand in 2004-05 is available from previous publications [12,17-20].

### Uncertainty in the number of deaths from RTI

An uncertainty analysis was done using Monte Carlo simulation with the Ersatz program (http://www.epigear.com, Brisbane, Australia). The VA cause attribution was validated with the subsample of deaths for which both a medical record and a VA interview were available (2,558 of 3,316) [12,17]. In this study, the corrected cause profiles were applied to total deaths using proportional mortality by age and sex. The sampling uncertainty of the redistributed causes of death was quantified using Monte Carlo simulation. Multinomial distributions were assumed for VA, medical record, and validation samples, and the distribution of causes of deaths was obtained by sampling simultaneously from the multinomial distributions. The misclassification matrix that governs the mapping from ill-defined to specific cause was assumed to have a Dirichlet distribution. However, other uncertainty, such as that around cause attribution in the medical records substudy, is not included.

### Life lost due to premature mortality

YLL was calculated by multiplying the mortality estimates by the GBD standard life expectancy for a death at each age [21]. Lognormal distributions were assigned around the number of fatal RTIs.

### Nonfatal RTI victims

To quantify the total number of nonfatal RTI victims, we used three national injury datasets. First, hospital data for 2004 were provided by the National Health Security Office (NHSO), which covers all hospitals under government authority. Second, the Bureau of Epidemiology, Ministry of Public Health, provided Injury Surveillance data (IS) from 1999 to 2004. Both datasets contain International Statistical Classification of Disease and Related Health Problems (ICD-10) codes for injury admissions (S00-T98 and V01-V98). Third, the Health and Welfare Survey data (HWS-2005), a survey of 19,914 households and 67,815 people, were obtained from the National Statistics Office (NSO). For our calculations, we used hospital data rather than IS because of its greater coverage. However, as the hospital admission database does not fully cover all hospitals in Thailand (largely missing private hospitals), we used the HWS-2005 survey to set an envelope of the total number of admissions based on the reported frequency of hospital admissions from all causes in the past year by type of hospital (community, provincial, tertiary or university, private, and other). Using inflation factors based on the HWS-2005 (Table 1), the results from the hospital admission database were extrapolated to the whole country in 2004. This resulted in estimates of i) the number of RTIs by age and sex; ii) causes and nature of injuries; and iii) the ratios between the numbers of deaths, admitted RTI victims, and nonadmitted RTI victims in IS and hospital data.

**Table 1:** Admissions and inflation factors by age, sex, and type of hospital

Age group and hospital	Number of hospital admissions				Inflation factors			
	
	HWS		Hospital data		Ratio*		Adjusted*	
	
	Male	Female	Male	Female	Male	Female	Male	Female
**0 to14 years**								
Community	276 125	207 069	358 046	28 916	0.77	0.73	1.00	1.00
Provincial	212 962	164 765	296 090	224 391	0.72	0.73	1.00	1.00
Tertiary †	87 970	53 076	12 096	9 299	7.27	5.71	7.27	5.71
**15 to 44 years**								
Community	362 841	858 609	262 897	573 504	1.38	1.50	1.38	1.50
Provincial	294 964	580 897	293 869	491 241	1.00	1.18	1.00	1.18
Tertiary†	176 276	228 963	13 817	20 310	12.76	11.27	12.76	11.27
**45 to 69 years**								
Community	372 887	450 715	273 227	344 354	1.36	1.31	1.36	1.31
Provincial	339 897	373 697	321 694	349 074	1.06	1.07	1.06	1.07
Tertiary†	87 950	94 643	11 320	13 134	7.77	7.21	7.77	7.21
**70+ years**								
Community	158 705	150 157	149 150	187 676	1.06	0.80	1.06	1.00
Provincial	149 654	170 890	158 631	179 756	0.94	0.95	1.00	1.00
Tertiary†	25 918	39 347	4 123	6 163	6.29	6.38	6.29	6.38

* Number of cases from HWS divided by number of cases from hospital data, 2004. Ratios less than 1 were assigned a ratio of 1.

† We included cases admitted to private hospitals (about 20% of hospitals in Thailand) in the tertiary hospital category.

#### Undefined external cause and nature of injuries

External cause (e.g., vehicle type) and nature of injury (body regions and consequences) were defined based on the 13 diagnosis codes in the hospital data for 2004. Each record has only one external code and one or more nature of injury codes. Records that had information only on the nature of injury but not on the external cause (13.9%) were proportionally redistributed to those that had information on external cause. Injury records that had an external cause code but no nature of injury code were assumed to represent late treatment and were excluded from the analysis to avoid double-counting.

#### Nonfatal RTI cases admitted to the hospital

Next, we determined the frequency of nonfatal RTIs classified into 32 body regions (based on the GBD method). This was done using variables that contained ICD-10 diagnosis codes for each RTI victim in the hospital data. Most injury cases had more than one diagnosis code. We applied a ranking by expected severity of the injury. For each record, the diagnosis ranked highest in severity was selected. The same method was used in the Thai burden of disease study 1999, the 2003 Australian burden of disease study, and the GBD study [11,22,23].

#### Long-term disability from RTIs

Two approaches were used to estimate the number of RTI victims with long-term disabilities. In our primary analysis, the number of nonfatal RTI admitted cases was multiplied by the proportion of RTI victims with any type of long-term disability as observed in our recent Thai study [24]. In this study, 4.6% of 9,013 nonfatal admissions due to RTIs from eight hospitals ended up with long-term disabilities. As these proportions varied by age, we used age-specific estimates. To enable comparison to estimates for 1999, we followed the conventional GBD model in a parallel analysis: the number of nonfatal RTI cases admitted to a hospital, by age group and sex, was multiplied by the proportions with long-term disabilities from the GBD model for each of 15 injury categories (Table 2).

**Table 2:** Parameters for calculating burden of road traffic injuries in Thailand, 2004

Injury categories	Thai data		GBD			
	
	Long-term burden		Long-term burden		Short-term burden	
	
	Proportion of disability	DW †	Proportion of disability	DW	Duration (day)	DW
Fractured skull (5^th^)	11	0.293	15	0.357*	39	0.431
Fractured face bones	0	-	0	-	43	0.223
Fractured vertebral column	0	-	0	-	51	0.266
Injured spinal cord (1^st^)	100	0.548	100	0.725	-	-
Fractured rib or sternum	0	-	0	-	42	0.199
Fractured pelvis	6	-	0	-	46	0.247
Fractured clavicle, scapula, or humerus	0	-	0	-	41	0.136
Fractured radius or ulna	0	-	0	-	41	0.180
Fractured hand bones	0	-	0	-	26	0.100
Fractured femur (6^th^)	10	0.229	5	0.272	51	0.372
Fractured patella, tibia, or fibula	0	-	0	-	33	0.271
Fractured ankle	0	-	0	-	35	0.196
Fractured foot bones	0	-	0	-	27	0.077
Other dislocation	0	-	0	-	7	0.074
Dislocated shoulder, elbow, or hip	0	-	0	-	13	0.074
Sprains	0	-	0	-	14	0.064
Intracranial injuries (2^nd^)	5	0.568	5	0.350	25	0.359
Internal injuries	0	-	0	-	16	0.208
Open wound	0	-	0	-	9	0.108
Injury to eyes	16	-	10	0.299*	7	0.108
Amputated thumb	100	-	100	0.165	-	-
Amputated finger	100	-	100	0.102	-	-
Amputated arm	100	-	100	0.257	-	-
Amputated toe	100	-	100	0.102	-	-
Amputated foot	100	-	100	0.300	-	-
Amputated leg	100	0.496	100	0.300	-	-
Crushing	0	-	0	-	34	0.218
Burns < 20%	-	-	100	0.001	30	0.158
Burns >20% and <60% (4^th^)	-	-	100	0.255	102	0.441
Burns >60% (3^rd^)	-	-	100	0.255	102	0.441
Injured nerves	31	0.065	20	0.064	-	0.064
Poisoning	-	-	0	-	3	0.609*
Average	5	0.575	-	-	-	-

Note: 1^st ^to 7^th ^is the rank of severity of injury to 32 body regions in the Global Burden of Disease study.

* An average DW because it varies by age.

† DW is reported if it contains more than 10 cases.

#### Nonfatal RTI cases not admitted to a hospital

The total number of nonfatal, nonadmitted RTI victims was obtained by applying a ratio of admitted injury victims to nonadmitted RTI victims from emergency department data. We found three Thai studies providing these ratios. First, the ratio from the IS data between 1999 and 2003 was 1:3.6. Second, RTI data in 2004 from three tertiary hospitals (Nakhon Si Thammarat in the south, Lampang in the north, and Ratchaburi in central Thailand) showed a ratio of 1:3.0. Third, data collected from five provinces for a cost-of-RTI study reported a ratio of 1:3.4 [25]. We used the average ratio of 3.3 nonfatal, nonadmitted RTI victims per admitted RTI victim to calculate the total number of nonfatal, nonadmitted RTIs in 2004. This does not account for people with potentially serious injuries who do not arrive at a hospital, but that number is likely to be low because health service access in Thailand is generally good as 95.5% of Thais are covered by health insurance [26].

We then classified all nonadmitted RTI victims by the 32 injury categories used in GBD studies by age and sex based on the pattern of RTI victim diagnoses in 2004 from the emergency departments of three tertiary hospitals representing three of the four regions of Thailand. Out of 43,000 injury records, about 23,000 were RTI victims, of which 17,000 were nonfatal and were not subsequently admitted. Records that had multiple diagnosis codes were analyzed with the same hierarchical method we used for RTI admissions.

#### Disability weights and proportions with long-term disability

To calculate long-term YLD, we multiplied the number of RTI victims with long-term disabilities calculated above by the average long-term disability weight (DW) of 0.57 that was found among those with permanent disabilities in a study we previously conducted in Thailand [13]. In that follow-up study, the loss of health-related quality of life was derived for 197 individuals with residual disabilities at six to 18 months after the injury. Their EQ-5D+ scores and a regression equation developed for the Australian Burden of Disease study were used to calculate a DW for each individual [27]. This equation was based on DWs and EQ-5D+ descriptors for 241 health states used in the Dutch Burden of Disease study. For estimates of DWs by specific injury categories, we assumed that health state valuations (i.e., 1-DW) for co-existing injuries combine multiplicatively [22,27] and fitted the data on individuals' DWs with data on the nature of their injuries by ordinary least squares regression [13]. In order to compare long-term YLD to the 1999 Thai burden of disease study, we also calculated long-term YLD using the same method used in 1999 with updated incidence and mortality data [11]. In both approaches, the short-term YLD was calculated using DWs obtained from the GBD dataset [23].

### Years lost due to disability

Estimating the number of YLDs required information on the duration of long-term disability, which equates with the life expectancy of the people with these disabilities. We distinguished two types of long-term disabilities: those that reduce life expectancy, and those that do not. The duration of long-term disabilities that we assumed do not carry a risk of death from complications, such as amputated limbs, was obtained directly from Thai life tables [17]. The duration of other long-term disabilities associated with an increased risk of death due to complications (including skull fracture, spinal cord injury, femur fracture, intracranial injury, burns, and injured nerves [27]) was calculated by multiplying mortality risks in the Thai life tables by relative risks of mortality as used in previous burden of disease studies [11,22]. These assumptions are supported more by expert judgment than by empirical evidence, but the impact on the overall results is likely to be small, given that YLD makes up only a small part of the overall RTI-related burden. Furthermore, variations in the relative risk of dying will only have a modest impact on YLD, partly due to the fact that health gains and losses in future years are subject to discounting. The duration assumptions for short-term disabilities were adopted from the GBD study (Table 2) [23].

#### Uncertainty analysis

We did an uncertainty analysis similar to the one for YLL described above. We assumed a Poisson distribution for the number of nonfatal RTI cases. The Thai average long-term DW was assumed to have a beta distribution and the proportion with long-term disability, a binomial distribution.

## Results

### Number of RTIs in Thailand

In 2004, road traffic crashes resulted in 567,000 victims in Thailand (Table 3). There were 24,800 (95% uncertainty interval (UI): 22,400-27,200) RTI deaths, equating to a crude RTI death rate of 40 per 100,000 people (95% UI: 36-44), 66 per 100,000 people in males (95% UI: 59-73) and 14 per 100,000 people in females (95% UI: 12-16). The age-adjusted death rate due to RTIs (using WHO's World Standard Population 2000-2025 [28]) was 39 per 100,000 people (64 and 14 for male and female, respectively). There were 126,000 hospital admissions and 417,000 emergency department visits for RTI.

**Table 3:** Number (rate per 100,000) of fatal and nonfatal RTIs in Thailand by age and sex, 2004

Age group	Type of RTI victims					
	
	RTI deaths*		RTI admissions		RTIs presenting at emergency departments	
	
	Male	Female	Male	Female	Male	Female
0-4	379 (18)	314 (16)	2 113 (103)	1 459 (76)	20 462 (997)	8 671 (449)
5-14	1 082 (22)	617 (14)	9 641 (299)	4 453 (98)	50 953 (1 056)	16 553 (363)
15-29	9 182 (119)	1 202 (16)	41 930 (544)	10 019 (134)	126 125 (1 637)	28 898 (386)
30-44	4 592 (57)	729 (9)	22 082 (274)	7 231 (87)	62 035 (769)	21 945 (263)
45-59	3 359 (64)	925 (16)	12 869 (246)	5 197 (92)	35 005 (669)	14 538 (257)
60-69	861 (49)	387 (19)	4 157 (238)	1 676 (84)	10 704 (612)	5 590 (279)
70-79	710 (74)	167 (14)	1 696 (177)	755 (61)	6 623 (690)	4 583 (371)
80+	225 (70)	79 (16)	349 (108)	183 (38)	2 108 (655)	1 778 (365)
Total	20 390 (66)	4 420 (14)	94 837 (307)	30 973 (98)	314 015 (1 016)	102 556 (323)

Note: *Adjusted fatal rate using World Standard Population was 39 per 100,000 people (64 and 14 for male and female, respectively).

### Burden of road traffic injuries

Thailand lost 673,000 DALYs (95% UI: 546,000-881,000) due to RTIs in 2004 (Table 4). The majority (88%) of DALYs lost were due to premature mortality. The RTI burden mostly affected men (82%) and was higher in men than in women at all ages. Of the 77,800 DALYs due to disability, 95% were from the long-term consequences. Young adults were disproportionately affected by RTIs; 69% of DALY loss occurred in victims aged 15 to 44.

**Table 4:** Burden of RTIs in Thailand from two models in 2004

Burden of RTI	GBD method with Thai data	Conventional GBD
	
	Median (95% UI)	Median (95% UI)
YLL		
Male	487 000 (374 000-699 000)	490 000 (374 000-699 000)
Female	106 000 (81 000-142 000)	106 000 (81 000-142 000)
Total	594 000 (469 000-805 000)	596 000 (469 000-805 000)
YLD short-term		
Male	2 880 (2 860-2 900)	2 880 (2 860-2 900)
Female	960 (950-970)	960 (950-970)
Total	3 840 (3 820-3 860)	3 840 (3 820-3 860)
YLD long-term		
Male	57 000 (43 100-68 900)	32 000 (32 000-33 000)
Female	16 400 (12 300-19 600)	10 800 (10 600-11 100)
Total	74 000 (55 400-88 500)	43 000 (42 700-43 600)
DALY		
Male	547 000 (434 000-758 000)	522 000 (409 000-734 000)
Female	123 000 (98 000-161 000)	118 000 (92 000-154 000)
Total	673 000 (546 000-881 000)	641 000 (515 000-852 000)

The crude rate of DALY loss was 10.8 per 1,000 people per year and 17.8 and 3.9 for males and females, respectively (Table 5). Standardized to the World Standard Population [28], these rates were 10.6, 17.2, and 4.1 per 1,000, respectively. The highest DALY rate was 21 per 1,000 at ages 15 to 29.

**Table 5:** Number of DALYs and DALY rate per 1,000 (in brackets) of fatal and nonfatal RTIs in Thailand by age and sex, 2004

Age	DALY						Total*	
			
	YLL		YLD					
			
			Long-term		Short-term			
			
	Male	Female	Male	Female	Male	Female	Male	Female
0-4	11 450 (5.6)	9 542 (4.9)	2 760 (1.3)	1 828 (0.9)	91 (0.04)	49 (0.03)	14 301 (7.0)	11 418 (5.9)
5-14	31 696 (6.6)	18 254 (4.0)	3 463 (0.7)	2 704 (0.6)	367 (0.05)	145 (0.03)	35 526 (7.4)	21 102 (4.6)
15-29	252 265 (32.7)	33 566 (4.5)	27 311 (3.5)	4 646 (0.6)	1 269 (0.16)	301 (0.04)	280 845 (36.5)	38 513 (5.1)
30-44	111 088 (13.8)	18 178 (2.2)	3 386 (1.7)	3 414 (0.4)	616 (0.08)	208 (0.02)	125 090 (15.5)	21 800 (2.6)
45-59	64 625 (12.4)	18 921 (3.3)	7 051 (1.3)	3 273 (0.6)	357 (0.07)	151 (0.03)	72 032 (13.8)	22 345 (3.9)
60-69	11 770 (6.7)	5 923 (3.0)	2 953 (1.7)	263 (0.1)	109 (0.07)	59 (0.03)	14 832 (8.5)	6 245 (3.1)
70-79	6 309 (6.6)	1 735 (1.4)	318 (0.3)	258 (0.2)	56 (0.06)	38 (0.03)	6 683 (7.0)	2 031 (1.6)
80+	982 (3.1)	410 (0.8)	150 (0.5)	- (0.0)	- (0.00)	- (0.00)	1 147 (3.6)	421 (0.9)
Total	490 185 (15.9)	106 529 (3.4)	57 392 (1.9)	16 385 (0.5)	2 864 (0.09)	950 (0.03)	550 456 (17.8)	123 876 (3.9)

Note: * The adjusted DALY rate using the World Standard Population was 10.6 per 1,000 (17.2 and 4.1 for male and female, respectively).

The use of Thai data on the average DW and the proportion of long-term disability for 2004 yielded almost double the amount of YLD in comparison to the estimate based on GBD assumptions. This difference in YLD was statistically significant but is small in comparison to the uncertainty estimated around YLL. Like mortality, YLD from long-term disability peaks at ages 15 to 29.

## Discussion

This study shows that the burden of disease due to RTIs in Thailand remained as large in 2004 as it was in 1999. The RTI fatality rate of 40 per 100,000 population was double the world average for low- and middle-income countries [2].

### Strengths

We used the best available RTI data for estimating the burden of RTIs in Thailand in an effort to avoid the limitations that often affect studies in low- and middle-income countries, such as underenumeration, limited reporting systems, and large uncertainties in the number of cases and deaths from specific causes of injury [5]. First, estimates of the number of fatal RTIs were obtained from the recent cause of death study [18,19]. This study reduced the number of deaths attributed to ill-defined codes and those missed through underregistration, major limitations of fatal RTI data in Thailand and other developing countries [5,16,29]. The number of fatal RTIs from cause of death studies was 1.8 times the report by the Royal Thai police in 2004. Second, we used recent local data to estimate the proportions and severity of long-term disabilities. Third, we included nonfatal, nonadmitted RTI cases. Though it increased the overall burden of RTIs only slightly, their inclusion allows the estimation of treatment costs that can be avoided by reducing RTI rates and thus facilitates economic evaluation of interventions.

### Limitations

Although we used the best available data, these data were not without flaws. In the absence of a nationally representative dataset, we had to estimate the number of nonfatal RTI victims by combining elements of different datasets. Comparison of different data sources (the IS and the hospital data) shows that the evidence on the magnitude of the RTI problem is inconsistent. This is likely due to differences in the reporting system, coverage, and quality of data in each dataset [11,30]. For example, hospital data in 2004 showed a decline in the total number of nonfatal RTIs since 1999. In 2004, 126,000 RTI victims were admitted to a hospital, down from 162,000 in 1999. In contrast, the Royal Thai police reported an increase in RTIs to about 14,000 fatal and 94,000 nonfatal cases in 2004, compared to 12,000 and 48,000, respectively, in 1999 [31], but this is likely due to a decrease in underreporting [32,33] and still well below our estimates.

The total number of deaths due to RTIs in this study is similar to the study in 1999 (24,413 cases), but 1.75 (UI: 1.45-2.01) times as high as figures reported in documents from the Ministry of Public Health [11,31]. These reports rely on mortality statistics in Thailand, which provide incorrect counts of cause of death, with large proportions attributed to "unknown causes" [16].

### Other studies

Premature mortality contributed the major part (88%) of DALY loss due to RTIs. This is a considerably higher proportion than estimated in Australia (72-73%), a Swiss canton (70%), Iran (62%), and Serbia (57%) [22,34-36]. An important reason for this higher proportion of fatal RTIs is the popularity of motorcycles, which are affordable for low-income families and young drivers [31,37]. In 2002, 68% of all vehicles registered in Thailand were motorcycles. In 1999, about 35% of all vehicle crashes involved motorcycles, and this increased to 45% in 2005. As a result, 65% (in 1999) and 82% (in 2005) of victims of fatal RTIs were motorcyclists [25,37]. The high fatality rates are partly because only 32% of motorcyclists wore helmets during the daytime and 9% at night, and only 30% of helmets comply with the Thai industrial standard [37,38]. An alternative explanation is that our study underestimates the nonfatal burden. However, the agreement between the hospital data and the data from the IS system suggests that our estimates are fairly accurate and that the high contribution of mortality to the RTI burden may be a specific Thai phenomenon.

Males, particularly those aged 15-29, are an extreme high-risk group as they contributed 47% (319,000 DALYs) to the total RTI burden in Thailand. This is similar to previous reports in Thailand estimating the male burden at three to five times the female burden, and similar to the situation in European countries (Austria, Denmark, Netherlands, Norway, England, and Wales) and Iran [7,30,39]. Thirteen percent (83,000 DALYs) of the burden of RTIs occurred in children less than 15 years old.

Results from a Thai follow-up study of long-term disability from RTIs led to a significantly higher estimate of YLD compared to an estimate based on conventional GBD assumptions. We used an average DW regardless of the nature of injuries. This approach is simpler and less error-prone than calculations that use estimates of the proportions of RTI victims with specific injury diagnoses and the related risks and severity of permanent injury. This empirically derived weight of 0.57 was higher than any of the GBD disability weights except for spinal cord lesion[13]. This higher average DW was the main cause of the difference in the long-term YLD as the average proportion of long-term disability was similar to that used in GBD studies [40].

The ratio of deaths (24,000) to admissions (126,000) and nonadmissions (417,000) of 1:5:17 in this study is similar to the ratio found in Bangladesh (1:8:28) [41]. It differs from the World Health Organization's ratio of 1:35:70 and raises questions about the use of global average data for specific countries [1].

### Implications

Our study reveals an apparent stagnation in the absolute size of the RTI burden between 1999 and 2004, despite a population increase of 3.9% over that period and increases in the number of vehicles from 6.3 million in 1999 to 16.6 million in 2002 and 26 million in 2007 [2,31,37]. This suggests that policy measures that have been implemented or intensified since 1999 may have prevented an increase in the burden of RTI. Such measures include general and sobriety checkpoints, raising public awareness of helmet and seatbelt use through mass media campaigns, speed limit enforcement, vehicle inspections, and traffic engineering, such as better road design [2,31,37]. It also suggests that greater efforts to reduce the burden of RTI in Thailand are urgently needed.

## Conclusion

In this study, the use of local data to calculate burden of RTI, rather than standard assumptions made in global and national burden of disease studies, made no difference to the results in general, but it provided much higher long-term YLD estimates. The method we used simplifies the estimation of long-term YLD because DWs specific to injury categories are not needed, in contrast to the standard GBD methodology. However, the proportions with long-term disability and the associated DW can only be used in populations that are similar to Thailand in terms of demographics, traffic, and the use of motorcycles. The availability of country-specific information on the frequency and consequences of health problems is a pre-condition for burden of disease studies. Although the situation in countries like Thailand is improving, too few studies are undertaken in low- and middle-income countries. These results will be valuable inputs to cost-effective analyses of interventions aiming to reduce the burden of RTIs.

## List of abbreviations

DALY: Disability-adjusted life year; DW: Disability weight; GBD: Global Burden of Disease; HWS: Health and Welfare Survey; ICD-10: International Statistical Classification of Disease and Related Health Problems 10^th ^edition; IS: Injury Surveillance; NHSO: National Health Security Office; NSO: National Statistics Office; RTI: Road traffic injury; UI: Uncertainty interval; YLL: Years of life lost due to premature mortality; YLD: Years lost due to disability;

## Competing interests

The authors declare that they have no competing interests.

## Authors' contributions

VD developed the framework of the study, did the analysis, and constructed worksheets to calculate the burden of road traffic injuries and estimate uncertainty, with input from LN, JB, MB, and TV. VD also drafted the manuscript. LV, JB, MB, and TV advised during data analysis and edited the manuscript. All authors read and approved the final version.
